# The circular RNA hsa_circ_0003091 regulates sepsis-induced lung injury by sponging the miR-149/Smad2 axis

**DOI:** 10.18632/aging.204125

**Published:** 2022-06-14

**Authors:** Mei-Jia Shen, Shen-Tao Yan, Xiao-Yan Zhang, Wen Li, Xu Chen, Xiao-Xiao Zheng, Guo-Qiang Zhang, Li-Chao Sun

**Affiliations:** 1 Emergency Department, China-Japan Friendship Hospital, Beijing 100029, China; 2 Graduate School of Peking Union Medical College, Chinese Academy of Medical Sciences, Peking Union Medical College, Beijing 100193, China; 3 Department of Respiratory and Critical Care Medicine, China-Japan Friendship Hospital, Beijing 100029, China; 4 Surgical Intensive Care Unit (SICU), China-Japan Friendship Hospital, Beijing 100029, China; 5 Department of Digestive, Beijing Ditan Hospital Capital Medical University, Beijing 100015, China; 6 Department of Emergency, Peking University People's Hospital (PKUPH), Beijing 100044, China

**Keywords:** acute lung injury, has_circ_0003091, Smad2, sepsis

## Abstract

Sepsis-induced acute lung injury (ALI) is a severe cause of death. Increasing evidence has identified circular RNAs (circRNAs) acting as critical regulators of human diseases. However, their expression pattern and underlying mechanisms in ALI remain unclear. Herein, we screened the circRNAs of ALI patients and constructed a lung injury murine model using lipopolysaccharides (LPS) induction. Functional analyses of targeted circRNA were performed *in vivo* and *in vitro*. Then, the downstream miRNA and mRNA of specific circRNAs were identified. Compared to healthy subjects, 35 circRNAs were upregulated and 9 circRNAs were downregulated in sepsis patients. The top 10 differentially expressed circRNAs were selected for validation and has_circ_0003091 was selected. The ALI mice presented significantly elevated has_circ_0003091 (mmu_circ_0015268). The functional analysis revealed that mmu_circ_0015268 contributed to the pulmonary injury, cell apoptosis, inflammatory responses, and endothelial activation in the ALI murine model. On the other hand, silencing mmu_circ_0015268 showed protective effects in LPS-treated mice and PMVECs. Furthermore, mmu_circ_0015268 sponged miR-149 to upregulate the expression of its target Smad2. In summary, we demonstrated that has_circ_0003091 might be a novel target for the management and treatment of sepsis-induced ALI.

## INTRODUCTION

Sepsis is caused by pathogen infections and is considered a serious and life-threatening disease with a high death rate. Acute lung injury (ALI) induced by sepsis is an acute disorder with respiratory failure [1]. During sepsis-induced ALI, dysfunction of epithelial permeability and microvascular leakage is induced by deregulated signals of apoptosis and inflammatory responses [2]. Thus, it is critical to identify the underlying mechanisms of vascular stability and permeability in sepsis.

Circular RNAs (circRNAs) are non-coding RNAs generated from protein-coding regions by back splicing [3]. Increasing studies have demonstrated that circRNAs are tightly involved in the regulation of mRNA stability by sponging microRNA (miRNA) in various human diseases [4, 5]. Recently, the expression profiles of circRNAs have been evaluated using an animal model of sepsis-induced ALI, and circRNAs/miRNAs interactions can be associated with inflammation, apoptosis, and mitochondrion distribution [6]. Additionally, another study evaluated the circRNAs or miRNAs profiles using whole-genome next-generation sequencing and identified potential pathogenic circRNAs or miRNAs in rat myocardial tissues after sepsis induction [7]. Moreover, the associations of different circRNAs with the endothelial permeability and vascular leakage were recently demonstrated [8, 9]. Particularly, circDNMT3B can contribute to vascular dysfunction in sepsis via miR-20b-5p sponging [10]. However, more circRNAs need to be identified to help in the prevention of sepsis and related organ injuries.

Therefore, in the present study, we for the first time characterized the profile of circRNAs in sepsis patients. Then, we functionally investigated their regulatory effects and underlying mechanisms using *in vitro* and *in vivo* models.

## RESULTS

### Differential expression of circRNAs in sepsis patients

**Figure 1:**
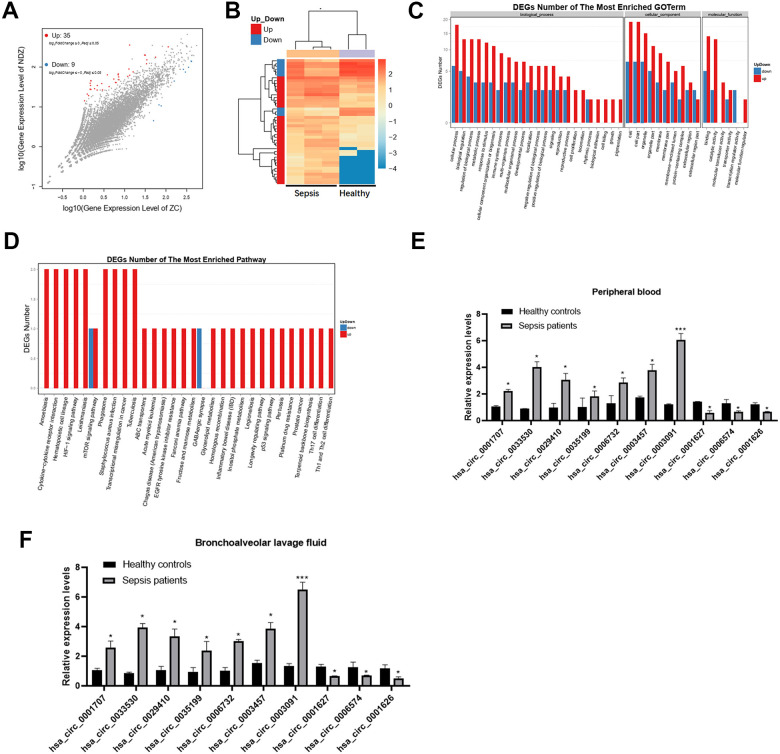
**Differential expression of circRNAs in sepsis patients.** (**A**) The scatter plot shows the circRNAs expression profiles between sepsis patients and healthy subjects. (**B**) Dysregulated circRNAs between sepsis patients and healthy subjects. (**C**, **D**) GO and KEGG enrichment analyses of dysregulated circRNAs between the two groups. (**E**, **F**) Validation of expression changes in top 10 circRNAs. *p < 0.05; ***p < 0.001.

First, we collected clinical blood samples from sepsis patients (n=3) and healthy controls (n=2) and performed transcriptomic profiling to identify deregulated circRNAs. A total of 44 circRNAs were differentially expressed (35 upregulated and 9 downregulated) between sepsis patients and healthy subjects (Figure 1A, 1B).

### Gene ontology (GO) enrichment and kyoto encyclopedia of genes and genomes (KEGG) pathway analysis of circRNA genes

Further, we found that upregulated circRNAs were associated with metabolic processes and catalytic activity in sepsis patients, while downregulated circRNAs were related to cellular processes (Figure 1C). The KEGG analysis revealed that cytokine-cytokine receptor interactions were associated with upregulated circRNAs, while fructose and mannose metabolism was related to downregulated circRNAs (Figure 1D).

### Validation of circRNAs expression by qRT-PCR

Consistent with the sequencing analysis, the qRT-PCR results revealed that, compared to healthy controls, 7 circRNAs were upregulated and 3 were downregulated in both peripheral blood and bronchoalveolar lavage fluid from sepsis patients (Figure 1E, 1F). Particularly, the expression of has_circ_0003091 was ranked top 1 and was selected for subsequent analyses.

### Expression of has_circ_0003091 in LPS-induced ALI mice

**Figure 2:**
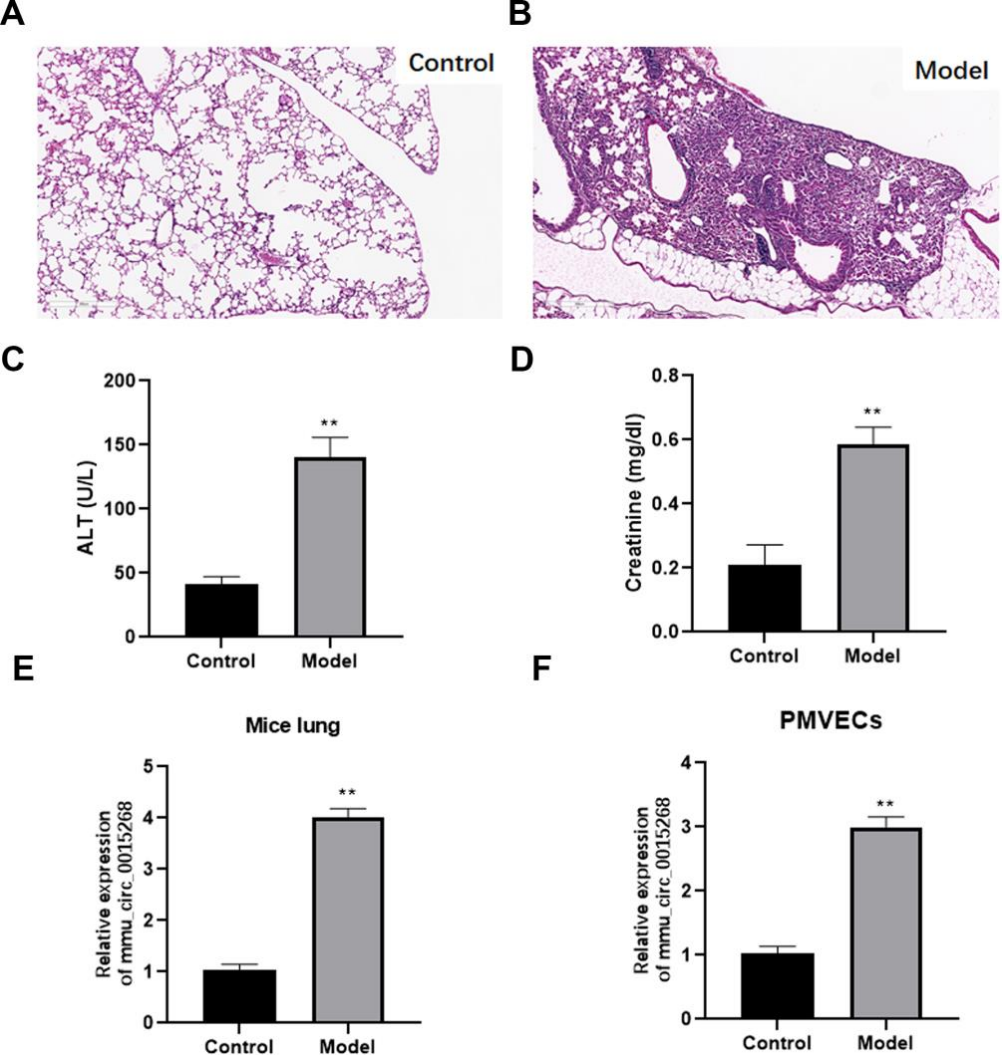
**Expression of mmu_circ_0015268 in LPS-stimulated lung tissues and PMVECs.** ALI model was evaluated by histological analysis (**A**, **B**), hepatic-injury marker (ALT activity) (**C**) and renal injury marker (creatinine concentration) (**D**). (**E**) Expression of mmu_circ_0015268 in whole lungs after LPS treatment. (**F**) Expression of mmu_circ_0015268 in primary lung microvascular endothelial cells (PMLECs) stimulated with LPS. **P < 0.01.

To explore the effects of has_circ_0003091 (mmu_circ_0015268) in endothelial cell dysfunction and pulmonary vascular injury, its expression was determined in the lung and lung EC tissues from LPS-induced ALI mice. The histological analysis showed that sepsis led to pulmonary injury (Figure 2A, 2B). Additionally, the levels of hepatic injury (ALT activity) and renal injury (creatinine concentration) markers were dramatically increased in sepsis mice compared to healthy controls (Figure 2C, 2D). These data indicated the successful establishment of the sepsis model. Compared to the lung tissues of healthy mice, ALI mice presented enhanced expression of has_circ_0003091 (Figure 2E). Meanwhile, we also observed significantly elevated expression of has_circ_0003091 in LPS-treated primary murine pulmonary microvascular endothelial cells (PMVECs) compared to untreated cells (Figure 2F). Altogether, these results showed that LPS induction significantly enhanced the expression of has_circ_0003091 in the lung and lung ECs tissues of ALI mice.

### Upregulation of has_circ_0003091 contributes to lung inflammation, endothelial dysfunction, and vascular injury

**Figure 3:**
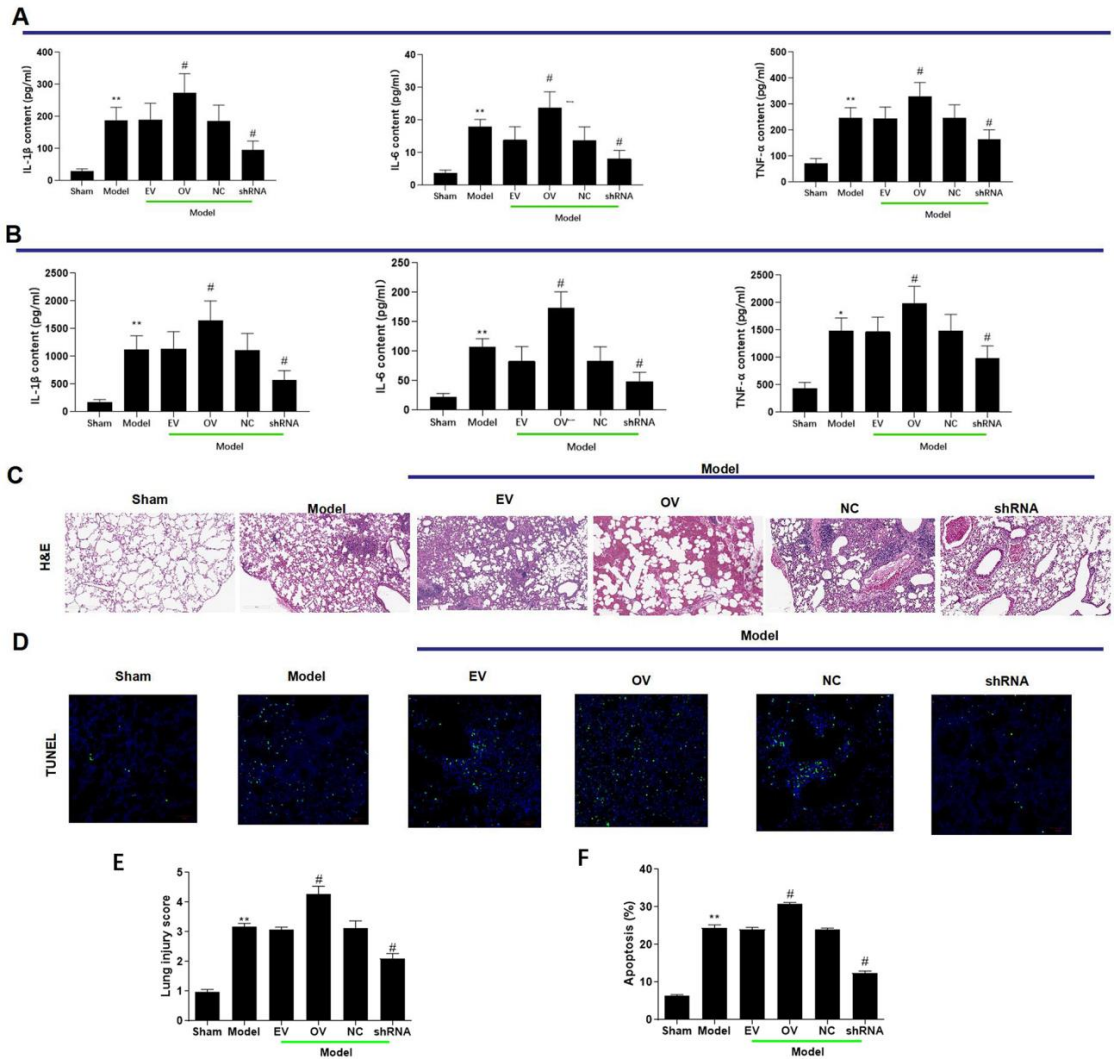
**mmu_circ_0015268 contributed to lung inflammation and vascular injury. ** Adult C57BL/6 mice were randomly assigned into 6 groups: sham, Model, Model + empty vector (EV), Model + mmu_circ_0015268 overexpressing vector (OV), Model + negative control (NC), Model + mmu_circ_0015268 shRNA (shRNA). ELISA was used to detect the contents of TNF-α, IL-6, and IL-1β in whole lung tissues (**A**) and BAL fluid (**B**). (**C**) Paraffin-embedded lung tissue samples were stained for H&E (magnification 200 X). (**D**) TUNEL assay of lung tissue sections for evaluation of apoptosis (magnification 200 X). (**E**, **F**) Quantification of lung injury scores and apoptosis. *P < 0.05; **P < 0.01 vs. sham; #P < 0.05, vs. model.

**Figure 4:**
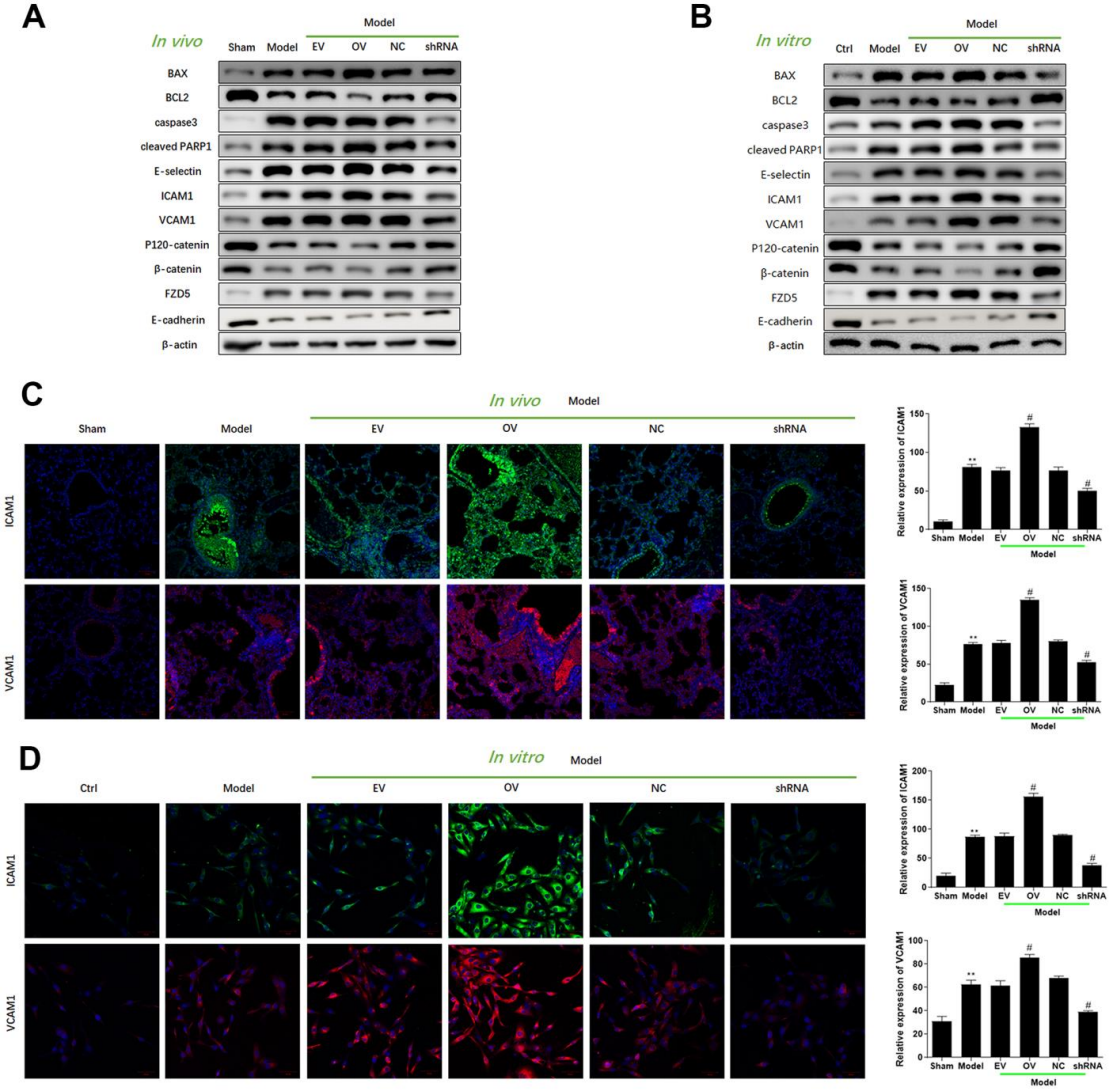
**Upregulation of mmu_circ_0015268 promoted endothelial dysfunction *in vivo* and *in vitro*.** (**A**) Representative blot results showing the levels of BAX, BCL2, Caspase3, cleaved PARP1, E-selectin, ICAM1, VCAM1, P120-catenin, β-catenin, and E-cadherin in lung tissues. (**B**) PMLECs were transfected with mmu_circ_0015268 overexpressing vector (OV) or mmu_circ_0015268 shRNA (shRNA), followed by LPS stimulation. Representative blot results showing the levels of BAX, BCL2, Caspase3, cleaved PARP1, E-selectin, intercellular cell adhesion molecule-1 (ICAM1), vascular cell adhesion protein 1 (VCAM1), P120-catenin, β-catenin, and E-cadherin in PMLECs. (**C**, **D**) Immunofluorescence intensities of ICAM1 and VCAM1 *in vivo* and *in vitro* for different groups.

Increased expression of has_circ_0003091 was associated with increased production of inflammatory cytokines in lung tissues, including IL-1β, IL-6, TNF-α (Figure 3A), and BAF (Figure 3B). The H&E and TUNEL stainings showed a higher pathological injury (Figure 3C, 3E) and apoptotic ratio (Figure 3D, 3F) of lung tissues in LPS-induced mice. Moreover, these changes were accelerated by has_circ_0003091 overexpression and alleviated by has_circ_0003091 suppression (Figure 3A–3F). Consistently, the protein levels of endothelial-specific adhesion marker E-selectin, ICAM1 and VCAM1, and apoptotic markers were highly induced by LPS in lung tissues (Figure 4A and Supplementary Figure 1). Further, significantly reduced levels of endothelial AJs proteins p120-catenin, β-catenin, and E-cadherin were observed in LPS-induced mice (Figure 4A), suggesting enhanced pulmonary vascular injury.

To verify the role of mmu_circ_0015268 on endothelial dysfunction, we transfected PMVECs with mmu_circ_0015268 overexpressing plasmids or mmu_circ_0015268 shRNA. Increased levels of ICAM1, VCAM1, and E-selectin were observed after has_circ_0003091 overexpression, suggesting aggravation of LPS-caused endothelial activation. Consistently, compared to LPS-treated control PMVECs, the LPS-treated PMVECs with has_circ_0003091 overexpression presented significantly reduced levels of AJs proteins p120-catenin, β-catenin, and E-cadherin, which was tightly associated with increased endothelial activation. On the other hand, mmu_circ_0015268 knockdown alleviated these damages (Figure 4B and Supplementary Figure 2). The quantitative evaluations of each protein are presented in the Supplementary Figures. Moreover, we applied immunofluorescence to detect the expression of ICAM1 and VCAM1 in LPS-treated animals and primarily cultured cells. Consistently, the intensities of ICAM1 and VCAM1 were enhanced in the model group, which was further enhanced by mmu_circ_0015268 overexpression and suppressed by mmu_circ_0015268 knockdown (Figure 4C, 4D). Collectively, these results indicated that mmu_circ_0015268 can promote lung injury responses in mice.

### mmu_circ_0015268 negatively regulates the expression of miR-149

**Figure 5:**
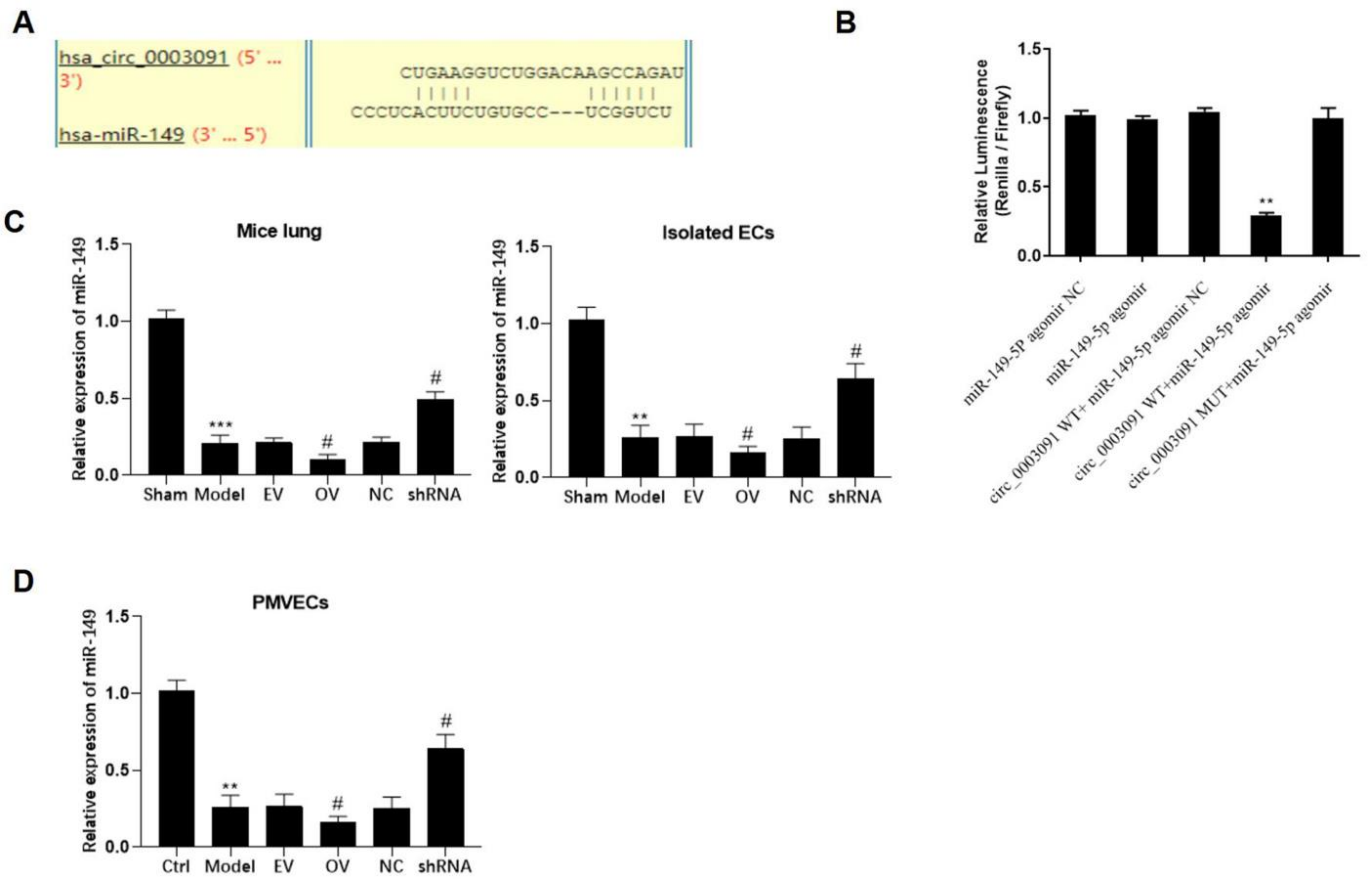
**mmu_circ_0015268 negatively regulated miR-149 expression.** (**A**) Putative binding site between has_circ_0003091 and miR-149. (**B**) Representative bar graphs showing the luciferase activity in the cells after indicated treatments. (**C**) Expression of mmu_circ_0015268 in lungs and isolated ECs. (**D**) The PMLECs were transfected with mmu_circ_0015268 overexpressing vector (OV) or mmu_circ_0015268 shRNA (shRNA), followed by LPS stimulation. The expression of mmu_circ_0015268 was measured in different groups. **p < 0.01; ***p < 0.001 vs. sham; ^#^*p* < 0.05 vs. model.

After bioinformatics analyses, miR-149 was identified as a potential target of has_circ_0003091 (Figure 5A). Compared to the mutant has_circ_0003091 cells, we observed significantly reduced luciferase activity in the miR-149 and WT has_circ_0003091 co-transfected cells (Figure 5B), suggesting the direct interaction between has_circ_0003091 and miR-149. Then, we examined the expression of miR-149 in lungs and PMVECs under ALI. The expression of miR-149 was significantly decreased in pathogenic lung tissues and LPS-stimulated PMVECs (Figure 5C). Compared to controls, has_circ_0003091 overexpression significantly impaired miR-149 expression in the mice’s lung tissues and isolated ECs (Figure 5C). Additionally, a negative correlation between miR-149 and has_circ_0003091 expression was observed in PMVECs (Figure 5D), consistent with the above results. Overall, these results suggested that has_circ_0003091 negatively regulates the expression of miR-149.

### Smad2 is a direct downstream target of miR-149

**Figure 6:**
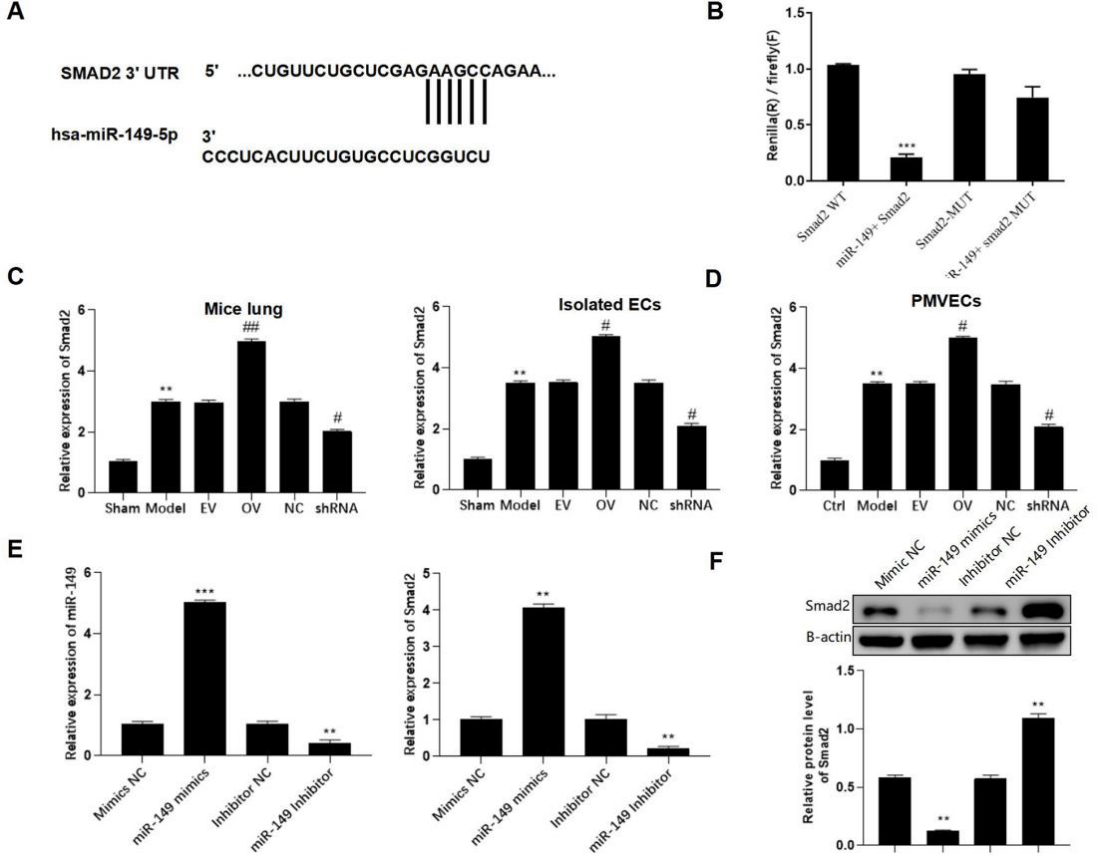
**Smad2 is a direct downstream target of miR-149.** (**A**) Putative binding site between Smad2 and miR-149. (**B**) Representative bar graphs showing the luciferase activity in the cells after indicated treatments. (**C**) Expression of Smad2 in lungs and isolated ECs. (**D**) The PMLECs were transfected with mmu_circ_0015268 overexpressing vector (OV) or mmu_circ_0015268 shRNA (shRNA), followed by LPS stimulation. Expression of Smad2 in different groups was measured. (**E**, **F**) The mRNA and protein levels of Smad2 in differentially treated PMVECs. **p < 0.01; ***p < 0.001 vs. sham; ^#^*p* < 0.05 vs. model.

The putative binding site between miR-149 and Smad2 was found using the TargetScan database and indicated that has_circ_0003091 exerted its regulatory function by sponging miR-149 and subsequently affecting the Smad2 expression in sepsis-induced ALI (Figure 6A). Additionally, after miR-149 mimic transfection, only Smad2-WT cells exhibited significantly reduced luciferase activity. No luciferase activity changes were observed in the Smad2-MUT cells, suggesting direct Smad2 targeting by miR-140 (Figure 6B). In the animal model, the levels of Smad2 in the lung tissues and isolated ECs were elevated (Figure 6C). Moreover, mmu_circ_0015268 could positively regulate Smad2 expression (Figure 6D). By transfecting PMVECs with miR-149 mimics or inhibitors, the mRNA and protein levels of Smad2 were significantly decreased or elevated, respectively (Figure 6E, 6F). These findings revealed that Smad2 was a downstream target of miR-149.

### mmu_circ_0015268 inhibits LPS-induced lung injury via the miR-149/Smad2 axis

**Figure 7:**
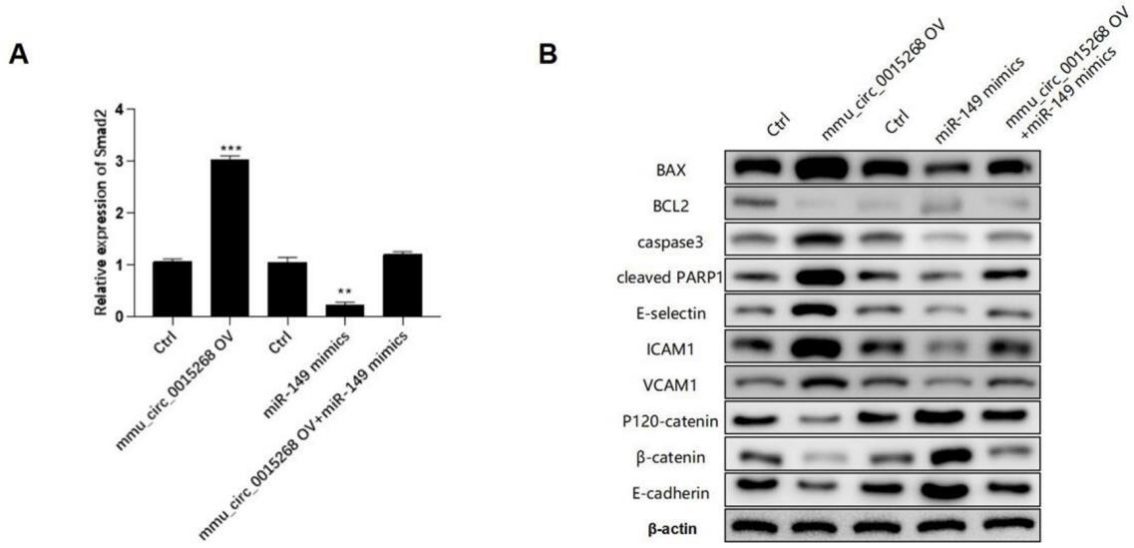
**mmu_circ_0015268 inhibited LPS-induced lung injury via the miR-149/Smad2 axis.** The PMLECs were transfected with mmu_circ_0015268-expressing vector and miR-149 mimics alone or combined, followed by LPS stimulation. (**A**) Expression of Smad2 in different groups. (**B**) Western blot of BAX, BCL2, Caspase3, cleaved PARP1, E-selectin, intercellular cell adhesion molecule-1 (ICAM1), vascular cell adhesion protein 1 (VCAM1), P120-catenin, β-catenin, and E-cadherin in different groups. **p < 0.01; ***p < 0.001.

The expression of Smad2 was significantly enhanced by has_circ_0003091 overexpression and impaired by miR-149 overexpression in LPS-stimulated PMVECs. Moreover, the elevated Smad2 expression was suppressed by miR-149 mimics (Figure 7A), suggesting that has_circ_0003091 exerted its function by regulating the miR-149/Smad2 axis. Furthermore, the Western blot analysis showed that mmu_circ_0015268-induced apoptotic markers and endothelial activation were effectively restored by miR-149 overexpression, including BAX, BCL2, Caspase3, cleaved PARP1, E-selectin, ICAM1, VCAM1, P120-catenin, β-catenin, and E-cadherin in PMVECs (Figure 7B and Supplementary Figure 3). These results suggested that mmu_circ_0015268 promoted LPS-stimulated apoptosis and inflammation via the miR-149/Smad2 axis in PMVECs.

## DISCUSSION

Acute respiratory distress syndrome (ARDS) or ALI is a devastating disease characterized by dysregulated immune responses, endothelial activation, and microvascular thrombosis [11]. Recently, several circRNAs have been identified to regulate the inflammatory response and cell apoptosis of pulmonary ECs through downstream miRNAs [12]. In the present study, we performed transcriptomic profiling to evaluate the expression of circRNAs in sepsis patients. We demonstrated that the has_circ_0003091/miR-149/Smad2 axis drives EC activation, inflammation, and apoptosis during ALI progression.

Through miRNA sponging, circRNAs can regulate the expression of target mRNAs [13]. Previously, Bao et al. evaluated the expression pattern of circRNAs in the lung tissues from CLP-induced ALI and identified 11 upregulated and 126 downregulated circRNAs [6]. In the present study, we collected clinical blood samples to screen deregulated circRNAs and found that 35 were upregulated and 9 were downregulated. Further, we selected has_circ_0003091 (ranked top 1 among deregulated circRNAs) for mechanistic studies. Several studies have performed in-depth functional analyses of deregulated circRNAs. For example, Zou et al. found that circRNAs (circ_0001679 and circ_0001212) mediated the protective effects of the P2X7R antagonist in sepsis-induced ALI by regulating the expression of Pln, Cdh2, and Nprl3 [14]. Jiang et al. demonstrated that circC3P1 attenuated the inflammatory response and cell apoptosis in ALI by modulating miR-21 [15]. Recently, exosomal circ-Fryl derived from ADSCs has been shown to play a protective role in sepsis-induced lung injury by regulating the miR-490-3p/SIRT3 axis [16]. However, the functional role of has_circ_0003091 (or mmu_circ_0015268) has not been previously reported and was identified for the first time in our current study.

Herein, we observed increased expression of mmu_circ_0015268 after LPS stimulation both *in vivo* and *in vitro*. The injection of mmu_circ_0015268-expressing adenoviral vector aggravated the inflammatory response, cell apoptosis, and endothelial activation in sepsis-induced ALI. Consistently, downregulation of mmu_circ_0015268 suppressed the elevated cell apoptosis and inflammatory cytokines production caused by LPA stimulation, suggesting the promotive effect of mmu_circ_0015268 on cell apoptosis and inflammation. Moreover, mmu_circ_0015268 over-expression aggravated LPS-induced endothelial activation, while its knockdown led to decreased expression of endothelial adhesion molecules and pro-apoptotic markers. In the current study, we explored the role of circ_0015268 on endothelial cell activation by using pulmonary microvascular endothelial cells to establish an ALI model. Nevertheless, the pathogenesis of ALI/ARDS also involves big damage to alveolar epithelial cells, which we will explore in the future. Altogether, these results demonstrated that mmu_circ_0015268 silencing might be a novel candidate for the treatment of sepsis-induced ALI.

Furthermore, it has been demonstrated that circRNAs can regulate gene expression by sponging miRNAs [17]. Consistently, we identified miR-149 as the target of mmu_circ_0015268. The expression of miR-149 was negatively regulated by mmu_circ_0015268. Moreover, miR-149 has been studied in various cancers, playing anti-carcinogenic roles by suppressing cell proliferation, migration, invasion, and inducing apoptosis [18–20]. Recent evidence has suggested that miR-149 over-expression can inhibit MyD88, TNF-α, IL-1β, and IL-6 levels [21]. Here, we showed that miR-149 overexpression restored the elevated apoptosis and endothelial activation caused by LPS stimulation in PMVECs. Thus, we further investigated the downstream target of the mmu_circ_0015268/miR-149 axis and found that Smad2 was directly targeted by miR-149. Smad2 is a receptor-regulated SMAD and serves as a transcriptional modulator. Previously, it has been reported that Smad2 plays a critical role in inflammatory responses, tissue differentiation, and cell apoptosis. Moreover, the TGF-β/Smad2 axis is a strong driver for tissue damage, such as lung fibrosis [22]. A recent study has reported that miR-7 regulated Smad2 activation and significantly participated in interstitial lung diseases [23]. Consistently, we observed significantly increased expression of Smad2 in ALI mice, and the delivery of mmu_circ_0015268 significantly elevated the expression of Smad2. Additionally, a negative correlation between miR-149 expression and Smad2 production was observed *in vitro*, suggesting the critical role of Smad2 in sepsis-induced ALI.

In summary, we demonstrated that has_circ_0003091 (mmu_circ_0015268) expression was upregulated in sepsis-induced ALI mice. Additionally, mmu_circ_0015268 silencing ameliorated sepsis-induced the production of proinflammatory cytokines, cell apoptosis, and endothelial activation via the miR-149/Smad2 axis. These findings indicated that has_circ_0003091 might be a novel target for sepsis-induced ALI treatment.

## MATERIALS AND METHODS

### Clinical samples

Blood samples were collected from three sepsis patients and two healthy subjects from the China-Japan Friendship Hospital following the Helsinki Declaration. Table 1 shows the demographic characteristics of septic patients. All samples were frozen in liquid nitrogen and stably stored at − 80° C until RNA extraction and sequencing at the Beijing Liuhe Huada gene technology company. The protocols and procedures of this study were strictly reviewed and approved by the Ethics Committee of the China-Japan Friendship Hospital. All subjects enrolled signed the informed consent.

**Table 1:** Demographic characteristics of septic patients.

**Characteristics**	**Septic patients (N = 3)**
**Sex**	
Male, n (%)	2(66.7)
Female, n (%)	1 (33.3)
Age, years	64± 8.7
Mortality, n (%)	1 (33.3)
**Comorbidities**	
Hypertension, n (%)	1 (33.3)
Chronic bronchitis, n (%)	1 (33.3)
Diabetes, n (%)	1 (33.3)
**Source of sepsis**	
Lung, n (%)	3 (100)
Mortality, n (%)	1 (33.3)

Data are expressed as number (%), mean ± SD.

### RNA sequencing

The PAXgene Blood RNA Kit (QIAGEN) was used to extract total RNA from clinical blood samples. A Nanodrop spectrophotometer (Thermo Fisher) was used to determine RNA concentration. Total RNA extraction and library construction were performed by the Annoroad Gene Technology Co., Ltd. (Beijing, China). The R software was used for subsequent data processing. The expression profile of circRNAs was screened based on the fold-change (FC) (FC > 2.0) and *p*-value (*p* < 0.05) thresholds. The genes related to the circRNAs screened were analyzed using GO and KEGG enrichment.

### Construction of sepsis-induced ALI model

First, C57BL/6 mice (10 weeks, 25-30 g) were randomly divided into 6 groups: Sham, Model, Model + empty vector (EV), Model + mmu_circ_0015268 overexpressing vector (OV), Model + negative control (NC), Model + mmu_circ_0015268 shRNA (shRNA). All mice were housed under a 12:12 h light/dark photocycle. LPS (0.5 mg/kg body weight) (Sigma) was used to induce sepsis via intravenous (i.v.) injection. An equal volume of sterile normal saline was used as vehicle control. Adenoviral vectors containing mmu_circ_0015268 cDNA or shRNA were packed into adenovirus particles, and i.v. injected one week before ALI induction. The animal study protocols were approved by the Institutional Animal Care and Use Committee of our hospital.

### Isolation of lung endothelial cells

After digestion with a mouse lung dissociation kit (Miltenyi Biotec), CD45+ immune cells were removed using the obtained CD45 single-cells conjugated microbeads. Then, CD31 conjugated microbeads were used to select endothelial cells. Finally, a suspension with CD45-negative and CD31-positive was selected.

### Cell culture

PMVECs were cultured using DMEM and transfected with mmu_circ_0015268 or mmu_circ_0015268 shRNA adenoviral vectors. After 24 h of transfection, cells were treated with LPS (100 ng/mL). Gene analysis was carried out 6 h after transfection.

### Enzyme-linked immunosorbent assay (ELISA)

Tumor necrosis factor-α (TNF-α), interleukin-6 (IL-6), and interleukin-1β (IL-1β) were quantified using ELISA kits (R&D Systems). After sample addition and 2 h of incubation with detection antibody, plates were incubated with a substrate solution and the optical absorbance at 450 nm was measured using a plate reader (Molecular Devices Corp, Menlo Park, CA).

### qRT-PCR

First, Total RNA was isolated from lung tissues or cells and reversely transcribed into cDNA. The expression of screened circRNAs was validated using qRT-PCR. Primers used in this study are shown in Table 2.

**Table 2:** Primers used for circRNAs.

**ID**	**Type**	**Primer**	**Product size**
hsa_circ_0001707	F	ACAGAGGCCTTCAACTTCCA	166
	R	GGGTTTTCATCTAGGAGGCG	
hsa_circ_0033530	F	AGACGTCCTCATCCAGCAG	235
	R	ACCCTTGTGTTCCTGAGCTT	
hsa_circ_0029410	F	AAGTACCGGTATTGGCCAGG	153
	R	CAATCAATCGTACCCCAGCG	
hsa_circ_0035199	F	CATTGTGCCTTTGGTCCTGG	216
	R	GGCTTTCACTATCCGTTCCAC	
hsa_circ_0006732	F	TCAGCCAAGAAAGACAACAAGA	252
	R	GGAAGCGGGAGATGTGAAAA	
hsa_circ_0003457	F	ACCAGCACATCAAAGGAAGC	170
	R	AGAAGAGAGGGCCAGTTGTG	
hsa_circ_0003091	F	TTTGCCAAGAGTCTAGCCCA	183
	R	TGGCCCACATCCATGATCTT	
hsa_circ_0001627	F	GGGTCTCACTCTGTCAACCA	168
	R	GTGCTTCAAGGGCTCATCAG	
hsa_circ_0006574	F	CCATTTCTGCCAACATCCCC	160
	R	CGGTTGCAGCCTTCAGATTT	
hsa_circ_0001626	F	TCAGAAAGTGAGGGCTCCAG	187
	R	GTGCTTCAAGGGCTCATCAG	

### Western blot analysis

Lysis Buffer and BCA Protein Assay Kit from Thermo Fisher were employed to extract total protein and determine their concentrations, respectively. After separation using 10% SDS-PAGE, protein bands were transferred to the PVDF membrane. After 1 h of blocking with 5% non-fat milk, samples were incubated overnight with primary antibodies against BAX (1:1000, Abcam, Boston, MA, USA), BCL2 (1:1000, Abcam, Boston, MA, USA), Caspase3 (1:1000, Abcam, Boston, MA, USA), cleaved PARP1 (1:1000, Abcam, Boston, MA, USA), E-selectin (1:1000, Abcam, Boston, MA, USA), ICAM1 (1:1000, Abcam, Boston, MA, USA), VCAM1 (1:1000, Abcam, Boston, MA, USA), P120-catenin (1:1000, Abcam, Boston, MA, USA), β-catenin (1:1000, Abcam, Boston, MA, USA), and E-cadherin (1:1000, Abcam, Boston, MA, USA) at 4° C. Finally, samples were incubated for 1 h with HRP-conjugated secondary antibodies and the protein bands were analyzed.

### Hematoxylin and eosin (H&E) staining

Changes in lung tissue morphology were determined using H&E staining. Briefly, lung tissue sections were stained with H&E following a routine protocol, and results were observed and recorded at 200 x magnification.

### TUNEL assay

The TUNEL assay (Promega, Madison, WI, USA) was used to determine cell apoptosis. Apoptotic cells were measured under a fluorescence microscope (200 x).

### Immunofluorescence

Briefly, tissue sections or PMVECs were washed with PBS, fixed with 4% polyformaldehyde, and penetrated with 0.5% Triton X-100. After 30 min of blocking with 5% BSA, sections were incubated with primary antibodies against ICAM1 and VCAM1 (Abcam) at 4° C overnight, followed by 30 min of incubation at 37° C with Alexa Fluor 488 and Alexa Fluor 555-labeled IgG. Then, DAPI staining was conducted for cell nuclei detection, and a light microscope was used to visualize the results.

### Dual-luciferase reporter assay

Lipofectamine 2000 was used to co-transfect HEK-293T cells with the luciferase report vector containing the 3’ UTR of WT-has_circ_0003091 or MUT- has_circ_0003091 and miR-149 agomir or agomir NC. After 24 h, the Luciferase Assay Reporter System (Promega) was used to evaluate the luciferase activity.

### Statistical analyses

The data were analyzed using GraphPad Prism 8.0 and are presented as means ± standard deviations (SDs). The data were compared using Student's t-test or ANOVA. A *p* < 0.05 was defined as a significant difference between indicated groups.

## Supplementary Material

Supplementary Figures

## References

[1] Rubenfeld GD, Caldwell E, Peabody E, Weaver J, Martin DP, Neff M, Stern EJ, Hudson LD. Incidence and outcomes of acute lung injury. N Engl J Med. 2005; 353:1685–93. 10.1056/NEJMoa050333 16236739

[2] Johnson ER, Matthay MA. Acute lung injury: epidemiology, pathogenesis, and treatment. J Aerosol Med Pulm Drug Deliv. 2010; 23:243–52. 10.1089/jamp.2009.0775 20073554 PMC3133560

[3] Chen LL, Yang L. Regulation of circRNA biogenesis. RNA Biol. 2015; 12:381–8. 10.1080/15476286.2015.1020271 25746834 PMC4615371

[4] Panda AC. Circular RNAs Act as miRNA Sponges. Adv Exp Med Biol. 2018; 1087:67–79. 10.1007/978-981-13-1426-1_6 30259358

[5] Lei K, Bai H, Wei Z, Xie C, Wang J, Li J, Chen Q. The mechanism and function of circular RNAs in human diseases. Exp Cell Res. 2018; 368:147–58. 10.1016/j.yexcr.2018.05.002 29730164

[6] Bao X, Zhang Q, Liu N, Zhuang S, Li Z, Meng Q, Sun H, Bai J, Zhou X, Tang L. Characteristics of circular RNA expression of pulmonary macrophages in mice with sepsis-induced acute lung injury. J Cell Mol Med. 2019; 23:7111–5. 10.1111/jcmm.14577 31411002 PMC6787439

[7] Zhang TN, Yang N, Goodwin JE, Mahrer K, Li D, Xia J, Wen R, Zhou H, Zhang T, Song WL, Liu CF. Characterization of Circular RNA and microRNA Profiles in Septic Myocardial Depression: a Lipopolysaccharide-Induced Rat Septic Shock Model. Inflammation. 2019; 42:1990–2002. 10.1007/s10753-019-01060-8 31332662

[8] Shan K, Liu C, Liu BH, Chen X, Dong R, Liu X, Zhang YY, Liu B, Zhang SJ, Wang JJ, Zhang SH, Wu JH, Zhao C, Yan B. Circular Noncoding RNA HIPK3 Mediates Retinal Vascular Dysfunction in Diabetes Mellitus. Circulation. 2017; 136:1629–42. 10.1161/CIRCULATIONAHA.117.029004 28860123

[9] Qin M, Wang W, Zhou H, Wang X, Wang F, Wang H. Circular RNA circ_0003645 silencing alleviates inflammation and apoptosis via the NF-κB pathway in endothelial cells induced by oxLDL. Gene. 2020; 755:144900. 10.1016/j.gene.2020.144900 32554046

[10] Liu J, Liu Y, Zhang L, Chen Y, Du H, Wen Z, Wang T, Chen D. Down-regulation of circDMNT3B is conducive to intestinal mucosal permeability dysfunction of rats with sepsis via sponging miR-20b-5p. J Cell Mol Med. 2020; 24:6731–40. 10.1111/jcmm.15324 32383354 PMC7299677

[11] Jiang J, Huang K, Xu S, Garcia JGN, Wang C, Cai H. Erratum to Targeting NOX4 alleviates sepsis-induced acute lung injury via attenuation of redox-sensitive activation of CaMKII/ERK1/2/MLCK and endothelial cell barrier dysfunction, Redox Biology 36 (2020) 101638. Redox Biol. 2021; 48:102200. 10.1016/j.redox.2021.102200 34844898 PMC8710995

[12] Yuan C, Gu J, Wu J, Yin J, Zhang M, Miao H, Li J. Circular RNA expression in the lungs of a mouse model of sepsis induced by cecal ligation and puncture. Heliyon. 2020; 6:e04532. 10.1016/j.heliyon.2020.e04532 32760833 PMC7393531

[13] Kristensen LS, Andersen MS, Stagsted LVW, Ebbesen KK, Hansen TB, Kjems J. The biogenesis, biology and characterization of circular RNAs. Nat Rev Genet. 2019; 20:675–91. 10.1038/s41576-019-0158-7 31395983

[14] Zou Z, Wang Q, Zhou M, Li W, Zheng Y, Li F, Zheng S, He Z. Protective effects of P2X7R antagonist in sepsis-induced acute lung injury in mice via regulation of circ_0001679 and circ_0001212 and downstream Pln, Cdh2, and Nprl3 expression. J Gene Med. 2020; 22:e3261. 10.1002/jgm.3261 32783373

[15] Jiang WY, Ren J, Zhang XH, Lu ZL, Feng HJ, Yao XL, Li DH, Xiong R, Fan T, Geng Q. CircC3P1 attenuated pro-inflammatory cytokine production and cell apoptosis in acute lung injury induced by sepsis through modulating miR-21. J Cell Mol Med. 2020; 24:11221–9. 10.1111/jcmm.15685 32846020 PMC7576301

[16] Shen W, Zhao X, Li S. Exosomes Derived from ADSCs Attenuate Sepsis-Induced Lung Injury by Delivery of Circ-Fryl and Regulation of the miR-490-3p/SIRT3 Pathway. Inflammation. 2022; 45:331–42. 10.1007/s10753-021-01548-2 34478012

[17] Huang A, Zheng H, Wu Z, Chen M, Huang Y. Circular RNA-protein interactions: functions, mechanisms, and identification. Theranostics. 2020; 10:3503–17. 10.7150/thno.42174 32206104 PMC7069073

[18] Sánchez-González I, Bobien A, Molnar C, Schmid S, Strotbek M, Boerries M, Busch H, Olayioye MA. miR-149 Suppresses Breast Cancer Metastasis by Blocking Paracrine Interactions with Macrophages. Cancer Res. 2020; 80:1330–41. 10.1158/0008-5472.CAN-19-1934 31911555

[19] Zhao L, Liu L, Dong Z, Xiong J. miR-149 suppresses human non-small cell lung cancer growth and metastasis by inhibiting the FOXM1/cyclin D1/MMP2 axis. Oncol Rep. 2017; 38:3522–30. 10.3892/or.2017.6047 29130108

[20] Lu H, Han X, Ren J, Ren K, Li Z, Sun Z. Circular RNA HIPK3 induces cell proliferation and inhibits apoptosis in non-small cell lung cancer through sponging miR-149. Cancer Biol Ther. 2020; 21:113–21. 10.1080/15384047.2019.1669995 31597523 PMC7012091

[21] Liang WJ, Zeng XY, Jiang SL, Tan HY, Yan MY, Yang HZ. Long non-coding RNA MALAT1 sponges miR-149 to promote inflammatory responses of LPS-induced acute lung injury by targeting MyD88. Cell Biol Int. 2019. [Epub ahead of print]. 10.1002/cbin.11235 31498515

[22] Mackinnon AC, Gibbons MA, Farnworth SL, Leffler H, Nilsson UJ, Delaine T, Simpson AJ, Forbes SJ, Hirani N, Gauldie J, Sethi T. Regulation of transforming growth factor-β1-driven lung fibrosis by galectin-3. Am J Respir Crit Care Med. 2012; 185:537–46. 10.1164/rccm.201106-0965OC 22095546 PMC3410728

[23] Zhang S, Jia X, Zhang Q, Zhang L, Yang J, Hu C, Shi J, Jiang X, Lu J, Shen H. Neutrophil extracellular traps activate lung fibroblast to induce polymyositis-related interstitial lung diseases via TLR9-miR-7-Smad2 pathway. J Cell Mol Med. 2020; 24:1658–69. 10.1111/jcmm.14858 31821687 PMC6991674

