# Supplementary material for: The circular RNA hsa_circ_0003091 regulates sepsis-induced lung injury by sponging the miR-149/Smad2 axis
Source: Aging (Albany NY). 2022 Jun 14;14(12):5059–74. doi: 10.18632/aging.204125 (PMC9271288; doi:10.18632/aging.204125)
Supplement: Supplementary Figures [file aging-14-204125-s001.pdf]

SUPPLEMENTARY FIGURES

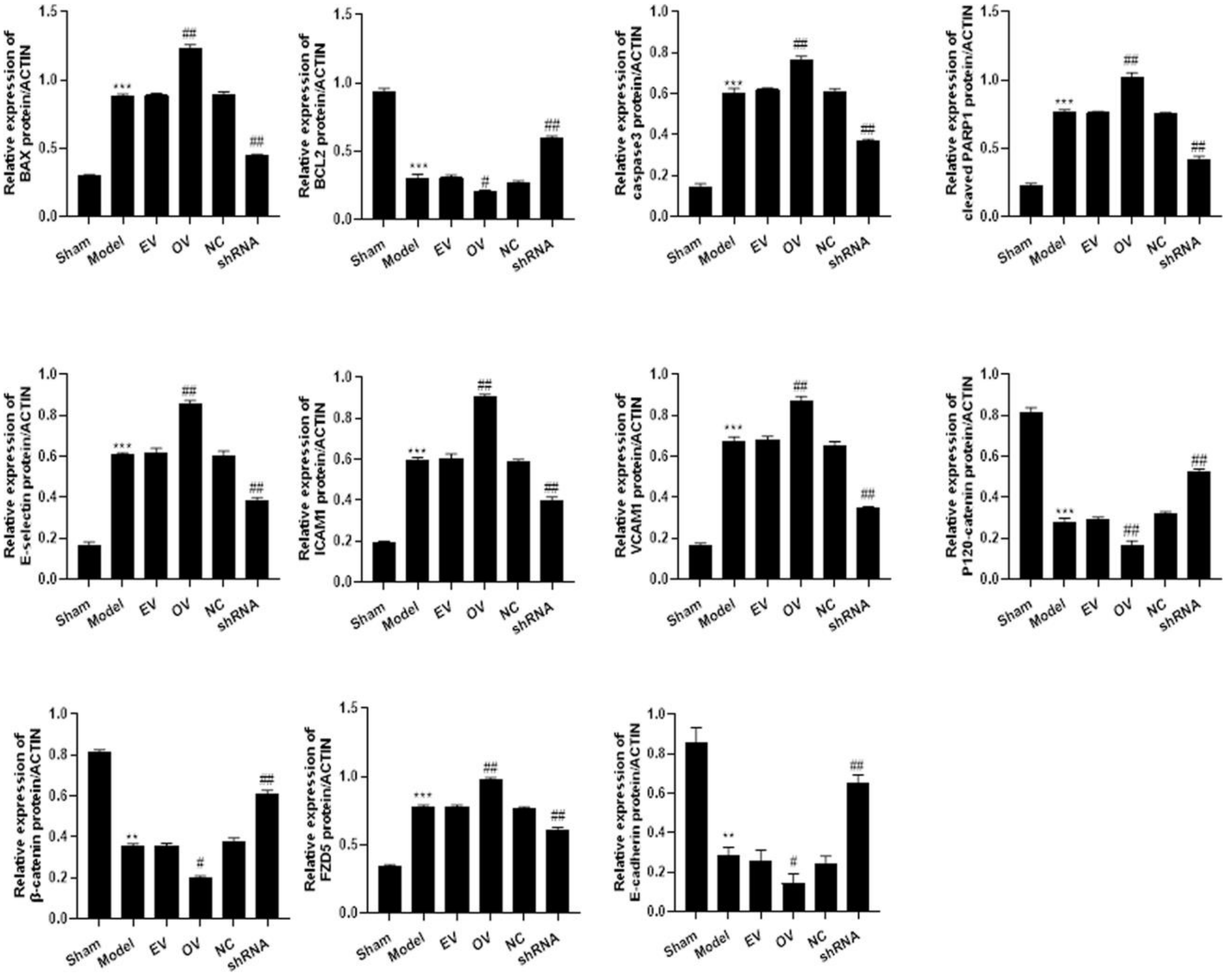

Supplementary Figure 1. Quantification of each protein level.

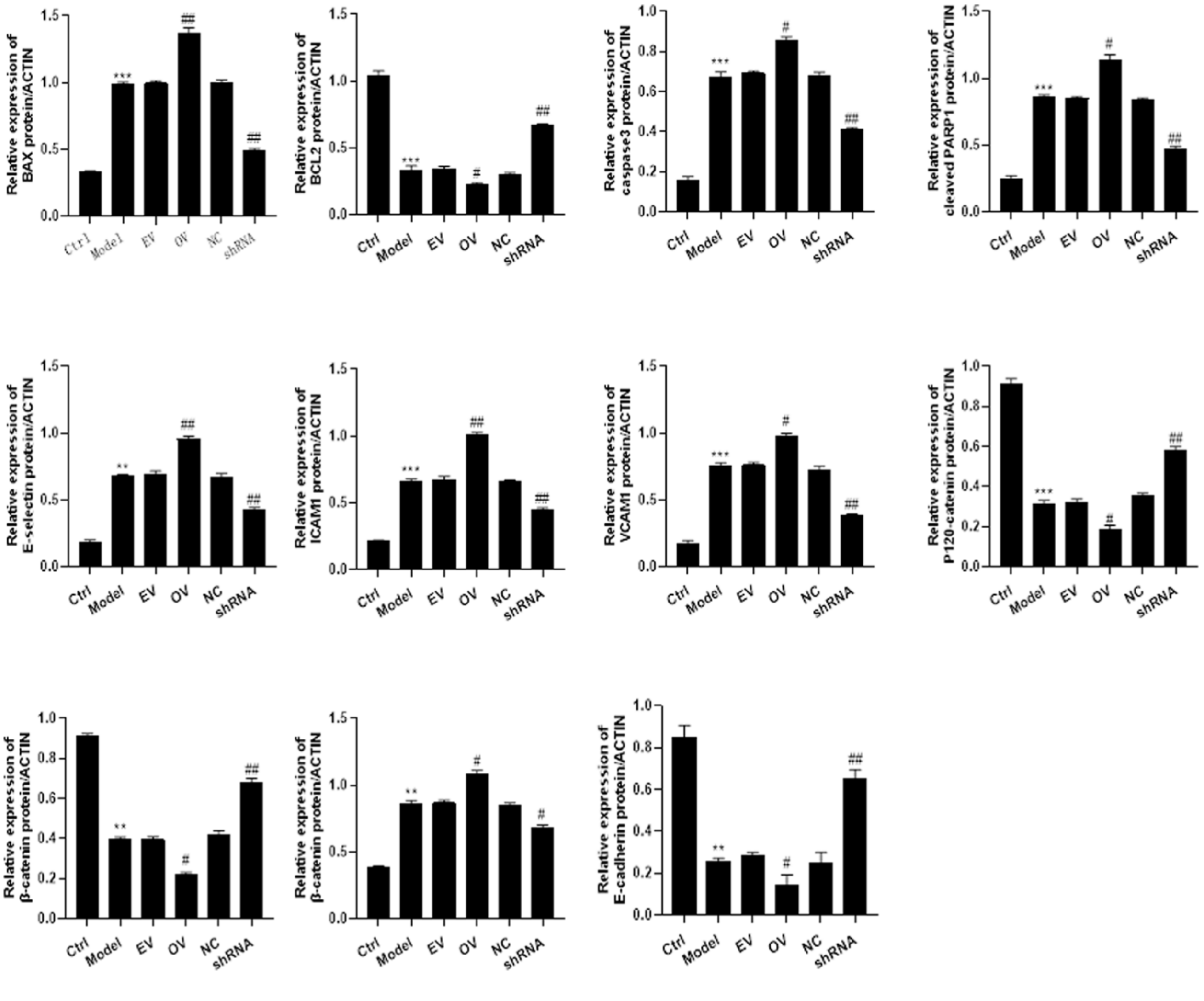

Supplementary Figure 2. Quantification of each protein level.

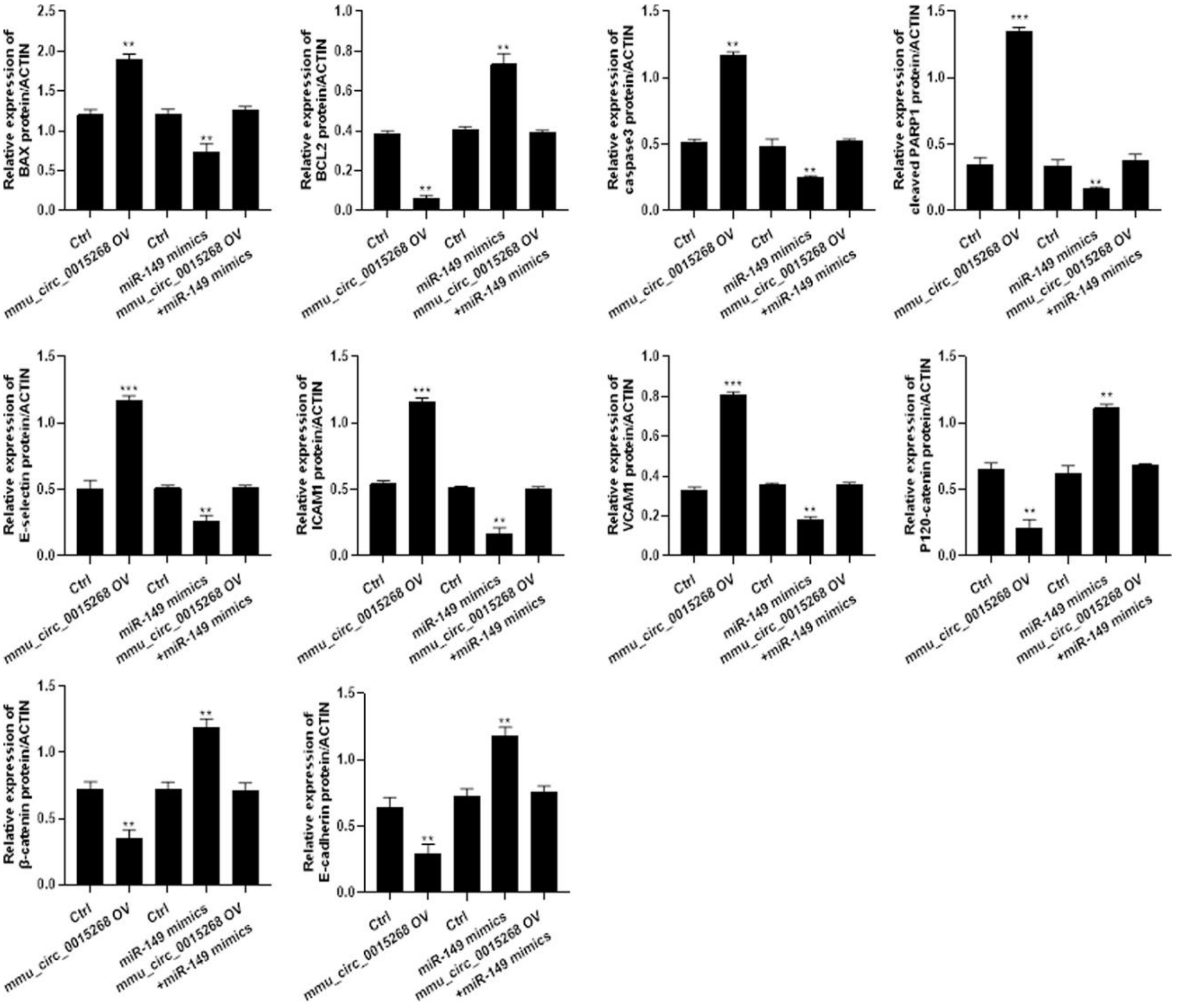

Supplementary Figure 3. Quantification of each protein level.
